# Evaluation of debridement effects of bromelain-loaded sodium alginate nanoparticles incorporated into chitosan hydrogel in animal models

**DOI:** 10.22038/IJBMS.2021.58798.13060

**Published:** 2021-10

**Authors:** Samaneh Bayat, Akram Rabbani Zabihi, Sara Amel Farzad, Jebrail Movaffagh, Ezzat Hashemi, Sepideh Arabzadeh, Maryam Hahsemi

**Affiliations:** 1 School of Pharmacy, Mashhad University of Medical Sciences, Mashhad, Iran; 2 Pharmaceutical Research Center, Pharmaceutical Technology Institute, Mashhad University of Medical Sciences, Mashhad, Iran; 3 Department of Pharmaceutics, School of Pharmacy, Mashhad University of Medical Sciences, Mashhad, Iran; 4 Department of Neurology and Neurological Science, Stanford University, Stanford, CA, USA; 5 Biotechnology Research Center, Pharmaceutical Technology Institute, Mashhad University of Medical Sciences, Mashhad, Iran; 6 Nanotechnology Research Center, Pharmaceutical Technology Institute, Mashhad University of Medical Sciences, Mashhad, Iran; 7 Department of Pharmaceutical Biotechnology, School of Pharmacy, Mashhad University of Medical Sciences, Mashhad, Iran

**Keywords:** Bromelain, Chitosan, Debridement, Nanoparticles, Sodium alginate

## Abstract

**Objective(s):**

Bromelain, a mixture of proteolytic enzymes from pineapple (*Ananas comosus*) is known as a potential debriding agent in burn treatment. In this research, the debridement efficiency of chitosan hydrogel loaded by sodium alginate-chitosan nanoparticles (NPs) containing bromelain (Br 10%-AG-CS NPs) was evaluated in animal models.

**Materials and Methods:**

The NPs were prepared using the ionic gelation technique and their properties were identified. Then, the debridement effect of bromelain NPs incorporated into chitosan hydrogel was evaluated 4 hr after wound treatment in animal models.

**Results:**

The particle size of positively charged Br-AG-Cs NPs was about 390±25 nm. The encapsulation efficiency of bromelain into AG-CS NPs was about 92%. The *in vitro* release profile showed that the maximum release of bromelain from NPs occurred during the first 4 hr (70%). The hydrogel structure did not significantly affect the profile release of bromelain in the formulation. After 6 months of storage at 4 and 25 °C, the synthesized NPs indicated no significant changes in bromelain activity. It was found that Br 10%-Ag-Cs NPs-CS hydrogel had the most beneficial effects on reducing necrotic tissues and resulted in re-epithelialization compared with other treated groups (negative and positive control, CS hydrogel, and Br 10%-CS hydrogel).

**Conclusion:**

Therefore, using this novel formulation can be considered a potential debridement agent.

## Introduction

Burn injury is a serious public health problem worldwide that is associated with substantial morbidity and mortality. The purpose of burn therapy is to improve the burn healing process and minimize the eschar, which is associated with increased risk of inflammation, infection, delayed healing, and scarring. Burn eschar removal (debridement) should be considered as the first crucial step of burn care (1-3). The surgical treatment of burn wound tissues is the most critical method of eschar removal. However, it can be associated with some problems, including pain, anesthetic risks, and high costs. Due to obstacles in this method, other techniques have been applied such as hydro-surgery, laser, mechanical debridement (wet to dry dressings), autolytic debridement (using occlusive dressings), biologic debridement (maggot therapy), and enzymatic debridement ( utilizing the collagenase) (3-5). Enzymatic debridement is an efficient method to remove necrotic tissue, reduce blood loss, and the rate of wound infection resulting in improved wound healing (3, 6, 7).

Bromelain, a raw herbal extract of pineapple, is rich in papain and cysteine protease with high proteolytic activity and good stability in high temperatures. Bromelain is used in many therapeutic applications, including inhibition of platelet aggregation, surgical traumas, enhancing the absorption of drug delivery, fibrinolytic, anti-inflammatory, anticancer, and wound healing debridement (3, 8, 9).

Recently, nanotechnology suggests new approaches for the treatment of acute and chronic wounds. The advantages of nano-systems include smaller and narrowing distribution of particle size, and appropriate physic-chemical properties led to enhanced drug penetration into the damaged tissue. 

They also protect the therapeutic agents from degradation and improve drug stability. In addition, the loading of drugs and biomolecules into nanocarriers enables various drug release profiles that can match the wound healing requirements. Different nano-delivery systems investigated for wound treatment include nanospheres, nanocapsules, nanofibers, nanoparticles (NPs), nanocarriers, nanoemulsions, and nanocolloids (10-12). 

Alginate has recently received more attention as a nanocarrier system in burn wound healing due to its biodegradability, biocompatibility, and ability to absorb water while maintaining its structure as well as controlled drug release (13-15).

Chitosan is a natural polysaccharide with appropriate properties in repairing skin such as antimicrobial, anti-inflammatory, hemostatic activity, and the ability to remove wound exudate, promote re-epithelization and accelerate angiogenesis (16-18). It was shown that chitosan could promote encapsulation efficiency and reduce the porosity of the alginate nanocapsules (19). 

The goal of this research is to evaluate the debridement effects of bromelain-loaded sodium alginate-chitosan NPs (Br-AG-CS NPs) incorporated into chitosan hydrogel on burn wounds in animal models. 

## Materials and Methods

***Experimental method***

*Material*

Bromelain, sodium alginate, and medium molecular weight of chitosan were obtained from Sigma Aldrich (Germany). BCA protein assay kit was purchased from Parstoos Company (Iran). Masson’s trichrome, hematoxylin and eosin staining were obtained from Merk (Germany). For anesthetizing the rats, ketamine 50 mg/ml and xylazine 2% were purchased from Trittau (Germany) and Alfasan Company (Iraq), respectively. 

***Preparation of Br-AG-CS NPs***

The ionic gelation technique was used to synthesize NPs as described previously with some modifications (17, 20). Firstly, 54 mg of sodium alginate was dissolved in 15 ml deionized water and stirred for 24 hr at room temperature. The pH of the alginate solution was adjusted between 5.2-5.6. Then, bromelain solution (54 mg in 3 ml deionized water) was added to the stirring sodium alginate solution followed by the addition of calcium chloride 0.3% w/v (6 ml). After 30–60 min of stirring, the solution was transferred into ice and sonicated with 90% power for 4 min. Next, 2.25% chitosan solution in acetic acid 1% (pH 4-4.6) was added drop by drop to the above solution, sonicated, and stirred for 1 hr. Finally, the synthesized NPs were washed with deionized water three times by centrifugation at 15000 rpm for 20 min and then re-suspended in water. 

***Characterization of synthesized NPs***

Determination of particle size and zeta potential of NPs was performed by a Dynamic Light Scattering device (DLS) (Nanozszen3600, Malvern). Briefly, 100 μl of the synthesized solution was suspended in 1 ml of deionized water and sonicated for 15 min in a water bath sonicator. Then, the size and Poly Dispersity Index (PDI) were measured by the mentioned device. Zeta potential measurement was done using the Zeta analyzer device by the Laster Doppler Velocity (LDV) technique. Microscopic observation of synthesized NPs was carried out using a Scanning Electron Microscope (SEM) (VP 1450 , Leo, Germany).

Examination of Fourier Transform Infrared spectroscopy (FTIR) was performed at the wavelength range of 4000-450 cm^-1 ^(DSC822e, Mettler Toledo, Switzerland). Via Differential Scanning Calorimetry (DSC), the thermal behavior of the solution was examined at the temperature range between 20–350 °C at a heating rate of 10 °C min^-^1 (DSC10, Nanjing Dazhan Institute of Electromechanical Technology, China). 

***Bromelain encapsulation efficiency***

The encapsulation efficiency of bromelain loaded into NPs was calculated using the indirect method. In the phase of NPs synthesis, after centrifuging, the supernatant containing unloaded bromelain was taken. After that, the quantity of bromelain was scaled by the BCA protein assay kit according to the manufacturer’s protocols. The encapsulation efficiency of bromelain was calculated by the conformance equation:

$\mathrm{Encapsulation efficiecy}\left(\%\right)=\frac{\mathrm{quantity of initial bromelain}-\mathrm{amount of unloaded bromelain}}{\mathrm{quality of initial bromelain}}\times 100$

***In vitro release study***

*In vitro* release of bromelain from NPs was performed in PBS solution, 0.1 M, pH 7.4. So, 1 ml of NPs suspension containing 50 mg bromelain was placed in dialysis cellulose membrane with 8000 Dalton cut-off and immersed in 5 ml PBS followed by constant shaking at the speed of 100 rpm at 37 °C. At predetermined times (1, 2, 3, 4, 24, 48, 72, 96, 120, 144, 168, and 192 hr), 300 μl of the released medium was removed and equally replaced with fresh PBS. The quantity of released Bromelain in samples was investigated using a BCA kit and the standard curve. 

***Enzymatic activity of bromelain loaded into NPs***

The enzymatic activity of bromelain was examined according to the assay as described before [21]. 1 ml of NPs was placed in dialysis tubing cellulose membrane with 8000 Dalton cut-off and immersed in 5 ml PBS. The samples were put in a shaker incubator at 37°C with permanent stirring. After 4 hr, 2.5 ml of gelatin 5%, warmed up to 80 °C, and then chilled up to 45 °C, was blended with 100 μl of released samples. Then, 10 μl of H_2_O_2_ 3% v/v was added to each sample and the pH was adjusted using NaOH 0.05 M, up to 6.9. After that, 1 ml of formaldehyde 37% v/v was added to each sample, and pH was moderated again using NaOH, up to 7.8. In the final step, the volume of needed NaOH (ml) represents the value of bromelain enzyme activity.

***Stability of enzyme activity***

To characterize the stability of enzyme activity of bromelain, the prepared solution (Br -AG-CS NPs) was stored at 4 °C and 25 °C for 6 months. After this time, 1 ml of NPs was placed in dialysis tubing cellulose membrane with 8000 Dalton cut-off and immersed in 5 ml PBS. The samples were put in a shaker incubator at 37°C with permanent stirring. 

***Preparation of chitosan hydrogel***

Chitosan (CS) hydrogel was prepared by dissolving 3 g of medium molecular weight of chitosan powder in 100 ml of acetic acid 1% v/v followed by stirring and sonication to remove air bubbles. 

***Loading of bromelain and NPs into the hydrogel***

For loading of bromelain into the hydrogel, 50 mg of bromelain was dissolved in a minimum amount of distilled water, added to 1 ml chitosan hydrogel 3% gradually and stirred for 2 hr. In addition, 1 ml of prepared Br-AG-CS NPs solution containing 50 mg bromelain was centrifuged at 15000 rpm for 20 min at room temperature. Then, the obtained NPs, re-suspended in 20 µl of deionized water, added to 1 ml of chitosan hydrogel 3% and stirred for 2 hr. 

***Viscosity measurement***

The determination of viscosity of chitosan hydrogel 3% w/v and chitosan hydrogel 3% w/v containing bromelain or Br-AG-CS NPs was gauged using a Brookfield R/S+ Rheometer (Brookfield Co., USA) rotational rheometer with CC25 spindle at a shear rate of 0-200 s-^1^.

***In vitro*** ***release study of bromelain loaded in hydrogel***

*In vitro* release profile of Br from hydrogel containing Br-AG-CS NPs was also carried out in PBS, pH 7.4. So that, 1 ml of chitosan hydrogel was transferred into the dialysis membrane. The profile release assay was carried out as described in *in vitro* release study section. 

***In vivo*** ***study***

For animal studies, 40 rats were considered at the weight range of 250–300 g. Each category was included in 5 groups (n=4): negative control, positive control (silver sulfadiazine), chitosan hydrogel, hydrogel containing bromelain10%, or Br 10%-AG-CS NPs. Then, the second burn degree on the rat’s skin was obtained using a flame heated metal coin with a diameter of approximately 2.5 cm. After burning, a sterile sodium hydrochloride 0.9% impregnated gauze, was put on the surface of the burn for 10 min. Then, 1 ml of each material was applied to the burn skin area of all rats. After 4 hr, the biopsies of wound regions were grabbed using a 1.5 cm biopsy punch and placed into 10% neutral formalin buffer. For pathological investigations, the prepared specimens of each biopsy were smeared with both hematoxylin and eosin (H&E) and also Masson’s trichrome. Biopsy samples were measured microscopically for studying the degree of burn and some other parameters such as re-epithelialization, permeation of inflammatory cells, necrosis, angiogenesis, and reproduction of fibroblasts and collagen deposition.

***Statistical analysis***

In order to analyze data, the statistical software GraphPad Prism 7 was used. By t-test and one-way ANOVA, the comparison between different groups was investigated. The Tukey-Kramer *post-hoc* test was used to compare the means of data in different groups. In this paper, *P*<0.05 was considered as significant level. 

**Figure 1:**
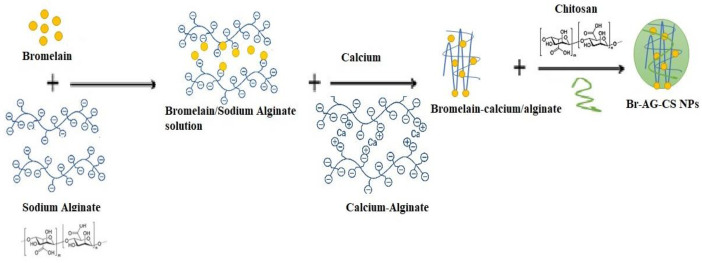
Scheme of synthesizing of Br-AG-CS NPs using ionic gelation method

**Figure 2:**
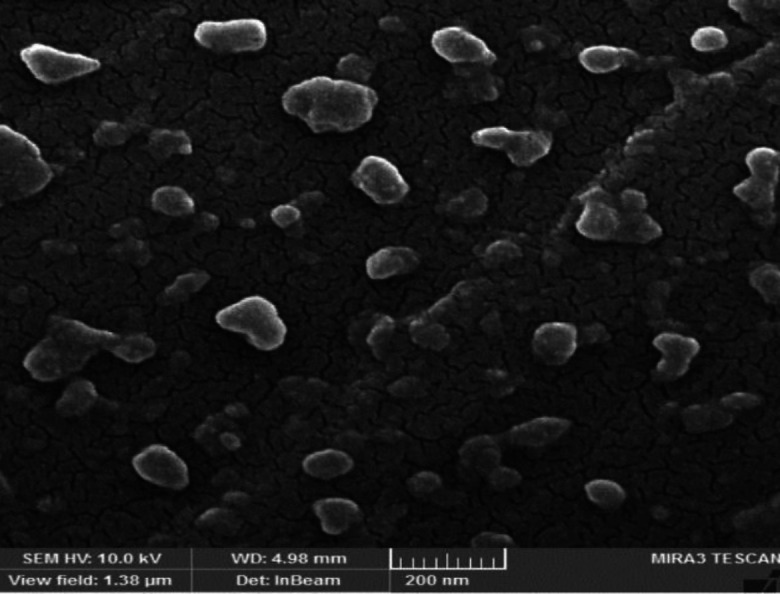
Morphological investigation of the prepared Br-AG-CS NPs using SEM

**Figure 3:**
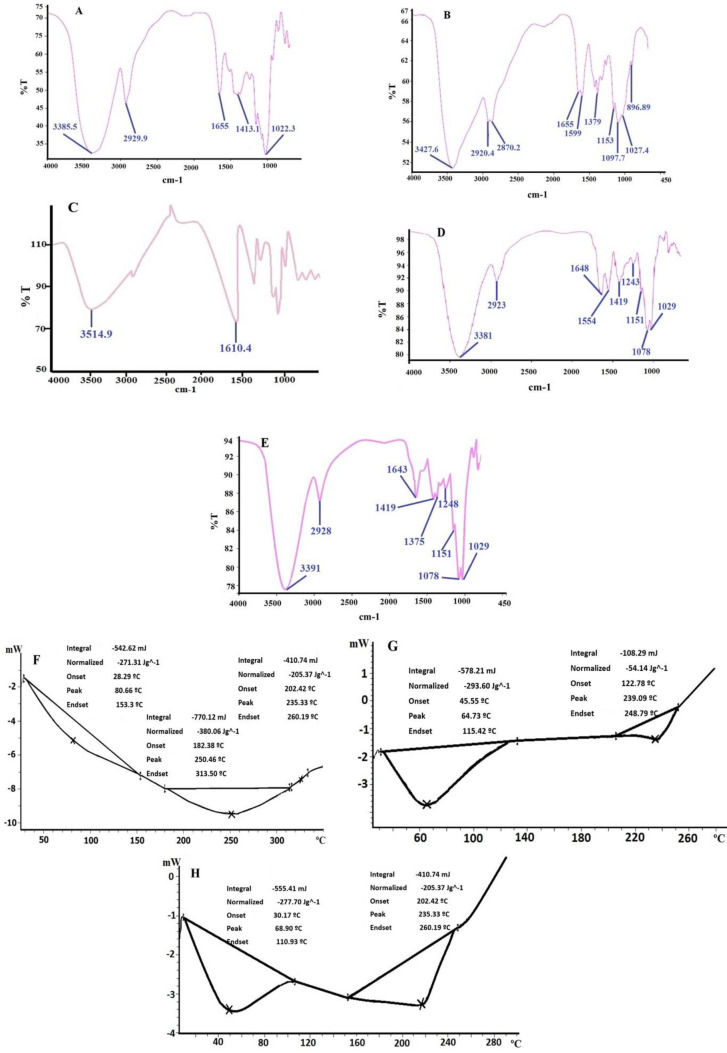
FTIR spectra analysis, A) Bromelain powder; B) Chitosan powder; C) Sodium alginate powder; D) Free NPs; E) Br -AG-CS NPs. DSC Analysis chart, F) Bromelain powder; G) Free NPs; and H) Br-AG-CS NPs

**Figure 4:**
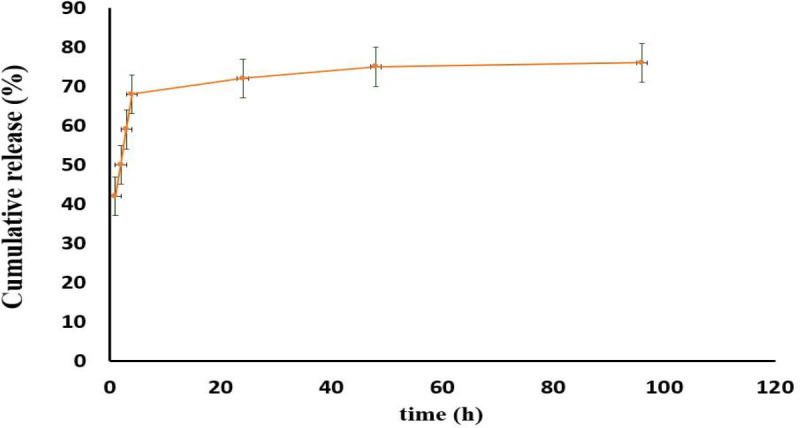
Release profile of bromelain from Br-AG-CS NPs in PBS (pH 7.4) at predetermined time. The quantity of released bromelain in samples was assayed using BCA kit

**Figure 5:**
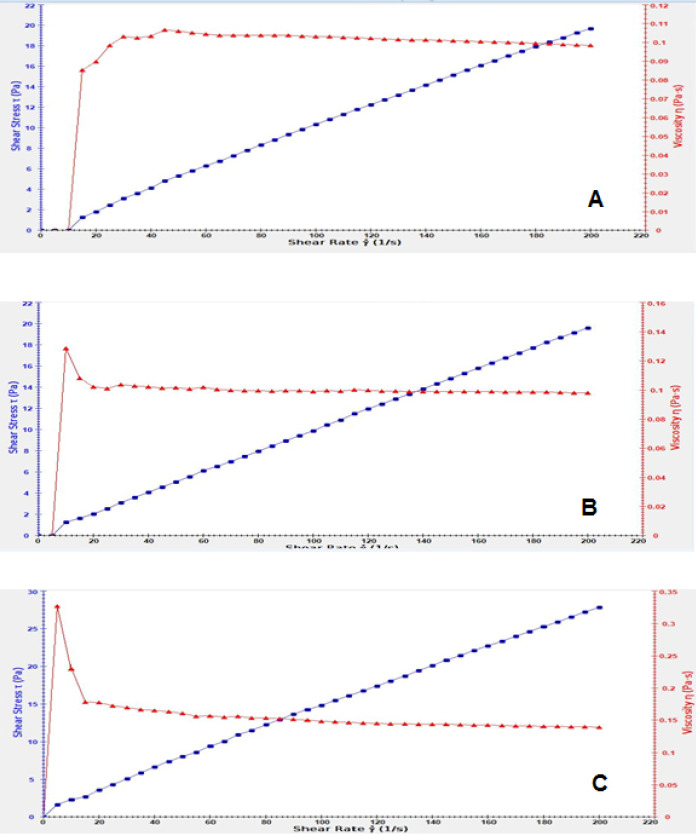
Viscosity related to hydrogels, A) CS hydrogel, B) hydrogel containing bromelain, C) Br 10%-AG-CS NPs-CS hydrogel. The red line shows the viscosity against the share rate and the blue line indicates the share rate/share stress

**Figure 6:**
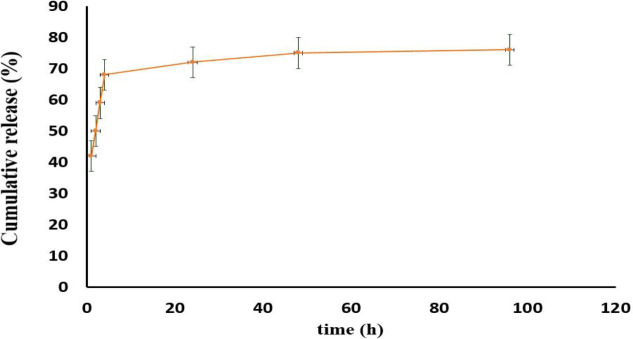
Release of bromelain from Br 10%-AG-CS NPs-CS hydrogel in PBS (pH 7.4)

**Figure 7:**
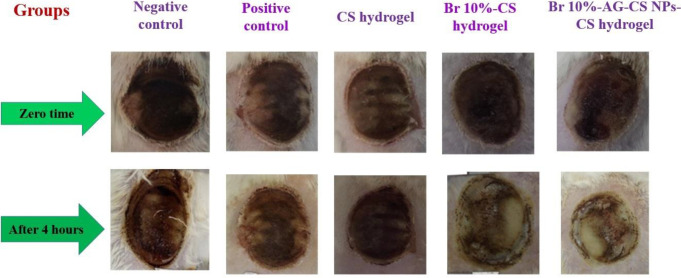
The vision of damaged tissues produced by a heated metal coin with a diameter of approximately 2.5 cm on the skin of rats and the regeneracy of skin at zero time and after 4 hr; In negative control, positive control, CS hydrogel, Br 10%-CS hydrogel, and Br 10%-AG-CS NPs-CS hydrogel groups

**Figure 8:**
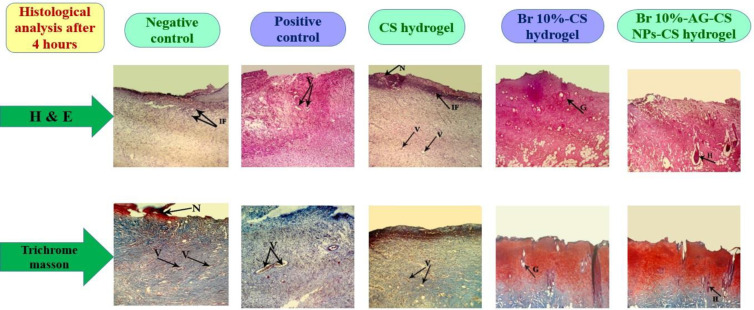
Histological study on damaged tissues after 4 hr. Using H & E staining and Masson’s trichrome; in negative control, positive control, CS hydrogel, Br 10%-CS hydrogel, and Br 10%-AG-CS NPs-CS hydrogel groups; magnification: ×100; N: necrotic tissue, IF: inflammatory cells, V: vessels, G: glands, H: hair follicle

## Results

***Synthesis of*** ***Br-AG-CS NPs***

In Figure 1, the schematic of bromelain loading in AG-CS NPs was shown.

***Characterization of NPs***

The particle sizes of Br-AG-Cs NPs and NPs without bromelain were obtained about 390±25 and 450±58 (PDI≤0.5), respectively. Zeta potentials were determined at 33.5±2.4 for NPs containing bromelain and 28±1.5 for NPs alone. SEM image also confirmed the formation of NPs, which were spherical or ellipsoidal (Figure 2).

The result of FT-IR analysis was shown in Figure 3. In the bromelain curve, N-H stretching and carbonyl groups corresponded to the bonds around 3385.5 cm^-1^ and 1655, respectively. The region of 1599 cm^-1^ in the chitosan graph corresponds to the N-H group and 3478 cm^-1^ confirmed the presence of O-H stretching (intramolecular hydrogen bond). The peak in the region 2877 and 2980 cm^-1^ is related to C-H symmetric and asymmetric stretching, respectively. In addition, the C=O bond of the first amide and C-N bond of the third amide were located in the region around 1100 cm^-1^ and 1379 cm^-1^, respectively. In sodium alginate also 3514 cm^-1^ belongs to O-H stretching and 2800 cm^-1^ corresponded to O-H as aliphatic. Stretching of carboxylate ion and pyranose ring was determined around the region 1610 cm^-1^ and 1100 cm^-1^, respectively.

Also, by comparing the FT-IR spectra, it was found that during loading of bromelain into NPs the peak of the N-H group in bromelain was shifted from 1655 cm^-1^ to 1643 cm^-1^. Furthermore, the region of the O-H group from 3381 cm^-1^ was moved to 3391 cm^-1^, which could be the reason for forming of an intermolecular bond between bromelain and NPs.

The loading of bromelain into NPs may shift the thermal properties, which was determined by DSC (Figure 3). The peaks of bromelain at 80.66 °C and 250.46 °C corresponded to water evaporation and melting point respectively as can be seen in Figure 3. Another peak at 325 °C was noted, which belonged to the destroying of the drug. In free NPs, two peaks were seen at 64.73 °C and 235.09 °C that corresponded to endothermic and exothermic reactions, respectively. By adding bromelain into NPs, the endothermic point shifted to 68.90 °C and exothermic point shifted to 235.33 °C which belongs to the melting point. According to the DSC results, it can be concluded that, by loading bromelain into NPs, all peaks in the bromelain curve decreased and shifted to the left. These alterations in peaks indicate the presence of bromelain in NPs and its effect on the thermal characterization of the NPs. 

***Loading analysis***

The loading efficiency of bromelain was determined using a BCA kit. The result showed that the encapsulation efficiency of bromelain into AG-CS NPs was about 92%.

***In vitro release study***

The *in vitro* release profile of bromelain from Br-AG-CS NPs was evaluated in PBS (pH 7.4) (Figure 4). The results indicated that the highest release of bromelain occurred during the first 24 hr, especially in the first 4 hr (70%). 

***Enzymatic activity of bromelain encapsulated into NPs***

The enzyme activity of bromelain loaded into NPs was obtained by the amount of NaOH 0.05 M, which required neutralizing H^+^ ions. It was observed that in 1 ml of Br-AG-CS NPs containing 50 mg of bromelain, the maximum activity of bromelain was about 96%. Therefore, loading of bromelain into AG-CS NPs did not affect its activity.

***Stability of activity***

This examination proved the stability of bromelain activity after 6 months of storage of Br-AG-CS NPs at 4 °C and 25 °C. According to the findings, bromelain had still 94% and 85% of the activity after the mentioned time, respectively.

***Viscosity measurement of chitosan hydrogel***

The result of this analysis showed that in all formulations when the shear stress increased, the viscosity decreased. Therefore, the hydrogels acted as pseudoplastic behavior. The highest reduction belonged to Br-AG-CS NPs-hydrogel (Figure 5). 

***In vitro*** ***release profile of bromelain loaded in hydrogel***

The outcome of *in vitro* release study of bromelain from Br-AG-CS NPs-CS hydrogel was evaluated in PBS (pH 7.4) (Figure 6). As it can be seen, the release pattern of bromelain from hydrogel is compatible with the pattern release of bromelain from NPs. It was determined that the hydrogel structure does not significantly affect the profile release of bromelain in the formulation.

***In vivo study***

In Figure 7, the appearance of burn tissues is shown in different groups after 4 hr of treatment. It can be seen that necrotic or dead tissues significantly decreased in treated groups with Br 10%-Ag-CS NPs-CS hydrogel.

The outcomes of histological examinations using H&E staining and Masson’s trichrome are shown in Figure 8. In groups of negative control, positive control, and only CS hydrogel, damaged dermis with epidermal detachment, necrotic tissue remnants, inflammatory cell infiltration, no hair follicles or sebaceous glands, and hyperemic vessels were observed. In contrast, in groups treated with Br 10%-CS hydrogel and in Br 10% -AG-CS NPs-CS hydrogel, the remaining dermis structure was preserved, along with a significant reduction in necrotic tissue and inflammation. Considering the microscopic examination, it seems that the Br 10%-AG-CS NPs-CS hydrogel group has the most beneficial debridement effect. 

## Discussion

In recent years, in order to enhance the procedure of burn wound therapy and also reduce the side effects of drugs, the use of NPs has attracted much more attention as a new drug delivery system (22, 23). The nano-carriers for topical administration should be non-toxic, biodegradable, and non-immunogenic and be able to release the drug in a controlled manner. The residence time, which is so important to decrease systemic absorption and adverse reactions, depends on the mucoadhesive properties, matrix, and size of the nano-carrier (23, 24). 

Recently, alginate has been used for topical wound dressing formulations including films, hydrogels, nanofibers, and NPs due to the ability to absorb the excess wound fluid and minimize the bacterial infections of the wound (25, 26). Chitosan is also one of the most useful polymers (27, 28) to prepare NPs and hydrogel applied in wound healing (29). A hydrogel is a cross-linked network of hydrophilic polymers that can absorb a large amount of water while maintaining the structure (30). In some types of research, the results indicated that the chitosan hydrogel could improve the process of tissue re-epithelialization in wound areas. Also, the fast wound closure and proliferation of epidermis cells were found in the treatment group by chitosan hydrogel (14, 31, 32). Honardar *et al.* demonstrated that the treatment of rabbits with second burn degree using chitosan-based gel caused the overgrowth of the epidermis with no hyperpigmentation. However, in the control group, an increase in the number of keratinocytes was observed due to the growth of papillary of the epidermis and also increased melanocyte pigmentation and melanin in the dermis (33). 

In this study, firstly, bromelain was loaded into AG-CS NPs. Bromelain is a proteolytic enzyme combination, which is known as a convenient debriding agent in burn wound healing and tissue regeneration. The increased stability of bromelain is an important consideration for its clinical application. Nano-formulation could protect the drug against denaturation and increase its biopharmaceutical properties. In different studies, AG-CS nano-carrier has been used to load different drugs for various therapeutic goals (13, 14, 34). The complex of AG-CS is formed by ionic reactions between the carboxylic group of alginate and amino groups of chitosan. The prepared NP is biodegradable, non-toxic, and biocompatible (35). 

The particle size of NPs is the main factor that can influence NP distribution, uptake, and efficiency in the body (36). In this research, the size of synthesized NPs containing bromelain was obtained at about 390±25 nm. In a study by Liu *et al.*, it was shown that skin infiltration and conservation of nitrofurazone NPs loaded into carbopol gel depended on the size of NPs. The maximum dissolution and permeability belonged to NPs with the lowest size (299 nm). On the other hand, the skin deposition was in the order of 611 nm > 299 nm > 906 nm. The final results demonstrated that the optimal size in this study was around 611 nm (37). 

The zeta potential of our synthesized NPs was positive. The amine groups of chitosan that are ionized under pH 6 were responsible for the positive zeta potential. Moreover, this positive charge caused the stability of the complex and prevented the accumulation of NPs. The positive zeta potential of NPs caused better penetration due to more interference with the surface (38). The NP size, which was measured by the nano-zetasizer device, was slightly larger than the micrograph quantitative analysis which was done by SEM. This difference is due to the contrast of SEM images. Such images just allow the center of NPs to be seen whereas the zetasizer device measures the radius around NPs [39]. According to the outcomes, the activity of bromelain did not change when it was loaded into NPs.

In many cases, drug release occurs according to some mechanisms such as release from the surface and due to erosion and diffusion from the swollen matrix (40). In this research, the maximum release of bromelain from NPs occurred during the first 24 hr especially at the first 4 hr (70%). This is probably due to dissolution and diffusion of trapped drugs in the AG-CS matrix. Slow and extended-release of bromelain may have led to the diffusion of drug and also degradation of matrix (14, 41, 42). Phosphate buffer medium was used due to 6.5–8.5 pH range of damaged tissues (43).

As the results showed, the chitosan hydrogel containing Br-AG-CS NPs had the appropriate viscosity. They behaved as pseudoplastic, which is found in solutions with medium concentrations of gums and natural and synthetic polymers. First in a stretched state and then in a rest position, it shows high consistency and viscosity. But after exposure to the skin and rubbing or stirring the hydrogel, its viscosity decreases and it acquires good pliability (29). It was determined that the hydrogel structure did not significantly affect the profile release of bromelain in the formulation.

Also, the stability of bromelain loaded into NPs was maintained after the specified period of time for 6 months, at 25 °C and 4°C. The consequences presented that there was no significant change in the bromelain activity after the mentioned time. In a study by Amid *et al.*, the enzymatic activity of recombinant and commercial bromelain was also evaluated using gelatin digestion unit assay at pH 4.5 and 45 °C. It became clear that bromelain in both forms had preserved its activity (44).

In different studies, the debridement effect of bromelain on burn wound healing has been evaluated [10, 11]. In the present research, it was found that Br 10%-AG-CS NPs-CS hydrogel, with a dose of 0.02 g/cm^2^ had the most beneficial effects on reduction of necrotic tissues and resulted in re-epithelialization compared with other treated groups. We also evaluated the wound healing process after 21 days (data not shown). In the recovered groups, developed epithelialization, increased amount of collagen, reduction in necrotic tissue, and mature granulation tissue were found, whereas, in the negative control group, no premature granulation and epithelialization tissue were observed, and scabbing persisted. In only the hydrogel group, thin re-epithelialization and premature granulation tissue were seen, and scabbing persisted. 

In some studies, the effect of enzymatic debridement of Debrase as a commercial bromelain product was studied on the lesion re-epithelialization. When the healing process by Debrase was compared with topical antibacterial agents and their carrier, the results showed faster re-epithelialization and cellular propagation [5, 6, 45]. In another research, Nexobrid as another commercial formulation of bromelain was topically applied for a maximum of 4 hr on the burn wound region, with a dose of 0.02 g/cm^2^. The clinical and non-clinical aspects were used for this commercial formulation. The results of the non-clinical aspect on animals were acceptable and showed that Nexobrid does not remove healthy tissues and fully removes eschars from the wound. The conclusion of the clinical trials study was performed according to GCP (Good Clinical Practice) and passed phase 2 trials. It is not advisable to use this combination for pediatric and patients who are more than 65 years old (46). In another paper, Nexobrid was used based on peer-reviewed publications and clinical relations. In order to re-evaluate, forty-three expert statements of Nexobrid were analyzed, and regarding the time of debridement, a classification was presented as: very early, early, and delayed in the following order respectively (12 hr, 12-72 hr, and more than 72 hr). In order to use Nexobrid, the updated guideline recommended indication, application, and post-interventional management and also, to prevent the pitfalls (47). In a study by Bayat *et al*., the effect of bromelain-chitosan nanofiber ‎was investigated on second degree burn in an animal model. The results indicated that this formulation could enhance the procedure of wound treatment at a dose of 0.06 mg/cm^2^ bromelain nanofiber (10). In another study, the proteolytic enzymes such as bromelain and papain, loaded into the chitosan gel, were applied for the healing of burns in rats. The results of these formulations showed earlier rate of re-epithelization, destruction of necrotic tissues, preventing infection, and reducing oxidation in the affected tissue compared with chitosan gel alone (48).

## Conclusion

Enzymatic debridement is an efficient method to remove necrotic tissue by contributing to improved wound healing. In this study, the chitosan hydrogel containing Br 10%-AG-CS NPs was prepared. The physicochemical properties of this drug delivery formulation were investigated and had favorable results. The debridement effect of hydrogel on 2^nd^ burn degree healing was also examined after 4 hr of exposure. The results suggest that this formulation accelerates the process of the removal of necrotic tissue in the animal model. Although it will need to be addressed in well-designed clinical studies, this delivery formulation can be recommended as a convenient debridement system for treatment of burns. 

## Authors’ Contributions

SB, ARZ, SAF, SA Study conception and design; SB, JM Data analyzing and draft manuscript preparation; EH Critical revision of the paper; MH Supervision of the research; SB, ARZ, SAF, JM, EH, SA, MH Final approval of the version to be published.

## Conflicts of Interest

The authors declare no conflicts of interest.
